# miR-33a-5p Targets RAP2A to Mediate the Sensitivity of Gastric Cancer Cells to 5-FU

**DOI:** 10.1155/2022/9701047

**Published:** 2022-08-22

**Authors:** Gang Ti, Zongliang Guo, Lili Li, Yongqiang Lv, Bin Yang, Jian Wang, Rui Guo, Yunqing Chen, Debin Meng, Feng Li

**Affiliations:** ^1^Department of medical record, Shanxi Bethune Hospital, Shanxi Academy of Medical Sciences, Tongji Shanxi Hospital, Third Hospital of Shanxi Medical University, Taiyuan, 030032 Shanxi, China; ^2^Department of General Surgery, Shanxi Province Cancer Hospital/Shanxi Hospital Affiliated to Cancer Hospital, Chinese Academy of Medical Sciences/Cancer Hospital Affiliated to Shanxi Medical University, Taiyuan 030012, China; ^3^Department of Radiotherapy Abdominopelvic, Shanxi Province Cancer Hospital/Shanxi Hospital Affiliated to Cancer Hospital, Chinese Academy of Medical Sciences/Cancer Hospital Affiliated to Shanxi Medical University, Taiyuan 030012, China; ^4^Department of Quality Control, Shanxi Province Cancer Hospital/Shanxi Hospital Affiliated to Cancer Hospital, Chinese Academy of Medical Sciences/Cancer Hospital Affiliated to Shanxi Medical University, Taiyuan 030012, China; ^5^Surgical VIP, Shanxi Cancer Hospital, Affiliated Cancer Hospital of Shanxi Medical University, Taiyuan 030012, China; ^6^Department of Cell Biology, Shanxi Province Cancer Hospital/Shanxi Hospital Affiliated to Cancer Hospital, Chinese Academy of Medical Sciences/Cancer Hospital Affiliated to Shanxi Medical University, Taiyuan 030012, China

## Abstract

**Objective:**

The objective of this study is to explore the effects of microRNA-33a-5p (miR-33a-5p)-ras-related protein Rap-2a (RAP2A) on biological functions of gastric cancer (GC) and to find the potential functional mechanism.

**Methods:**

We measured the miR-33a-5p expression in 30 GC tissues and cellular level and 30 adjacent normal tissues as control. Besides, the expression of miR-33a-5p was checked at cell level as well. To screen the possible targets of miR-33a-5p, prediction software was used and gene RAP2A attracted our attention. Through a series of experiments including real-time polymerase chain reaction (qRT-PCR), luciferase assay, and western blotting (WB), we verified RAP2A as a potential target of miR-33a-5p. The impacts of miR-33a-5p and RAP2A on biological functions of GC cell lines (BGC-823 and MGC-803) were analyzed by subsequent experiments. Cell invasion was tested by invasion assays. Cell proliferation was measured by cell counting kit-8 (CCK-8) assay. Cell clone was measured by clone formation assays. Finally, the expression of RAP2A protein was analyzed by WB assay.

**Results:**

We found miR-33a-5p was expressed lowly in GC tissues and cells. Overexpression of miR-33a-5p in BGC-823 and MGC-803 cells greatly inhibited the cell invasion and colony number. Furthermore, compared to sh-control (shControl), RAP2A knockdown (sh-RAP2A/shRAP2A) raised the sensitivity of GC cells to 5-FU significantly, characterized as reducing cell apoptosis.

**Conclusions:**

The expression of miR-33a-5p was lower in GC cell lines and tissues obviously, indicating that miR-33a-5p served as the antitumor gene in GC. The expression of RAP2A regulated negatively the sensitivity of GC cells to 5-FU. According to our in vitro experiments, miR-33a-5p/RAP2A was likely to become a new therapeutic target for GC.

## 1. Introduction

Gastric cancer (GC) is one of the most common malignant tumors in the world and the third leading cause of cancer-associated mortality worldwide [1]. The early diagnosis and treatment rate of GC are not high, both less than 10% in China [2]. However, the incidence and mortality of GC remain high. The 5-year survival rate is not optimistic [3]. Previous studies showed that GC development was relevant to many other factors, including familial inheritance, living habits, Helicobacter pylori infection, and other pathogenic reasons such as chronic atrophic gastritis and environmental factor [4, 5]. Due to the lack of specific symptoms in the early stage of GC, it has already advanced to the middle and late stages when detected, which is the main reason for the poor prognosis of most patients [6]. Therefore, there is an urgent need to find new diagnostic and therapeutic approaches to reduce GC-related mortality and improve patient clinical outcomes.

microRNAs (miRNAs) have 18-22 nucleotides and not encode RNA. But they can regulate the expression through the 3′UTR region of complementary mRNA [7, 8]. Many studies have found that miRNAs are involved in regulating many biological processes such as cell growth and apoptosis [9, 10]. In particular, differential expression of miRNA is involved in the development of some tumors [11]. For example, miR-33a-5p blocks the progression of esophageal cancer by KK1-Wnt/*β*-catenin axis [12]. PD-L1 inhibitor can regulate the miR-33a-5p/PTEN axis and increase the sensitivity of glioblastoma to radiation [13]. Meanwhile, Yanfen Lian et al. found that miR-33a-5p-RAP2A inhibited pancreatic duct adenocarcinoma cell growth. Furthermore, Dan Lin et al. and Qiong Wu et al. explored that the expression of miR-33a-5p was descending in GC. However, how it regulates drug resistance of target genes was rarely studied [14, 15].

Rap2A is a member of the RAS oncogene family. RAP2A gene, located at 13Q34, contains a 549 bp open reading frame with a molecular weight of 20,434 and can encode 183 amino acids [4]. Studies have revealed that RAP2A can regulate cell migration, cytoskeletal recombination, and other cellular processes [5–7]. In addition, it has been reported that RAP2A is upregulated in prostate cancer, thyroid cancer, nasopharyngeal cancer, and other human cancers [8–10]. However, the expression of RAP2A in GC has not been reported, except for one study that detected high expression of Rap2A in human hepatoblastoma HepG2 cells.

The relationship between GC and ncRNA has been proved by many studies, but the function and mechanism still needs to further explore. MiR-33a-5p has a regulatory role in many tumors, including GC. Thus, our study speculated the occurrence and development of GC by regulating miR-33a-5p/RAP2A and verify it through relevant experiments. The flow cytometry and CCK8 assay confirmed that miR-33a-5p can inhibit GC cell apoptosis and proliferation. Furthermore, dual luciferase reporter assay confirmed that RAP2A may target miR-33a-5p. RAP2A was upregulated in GC and modulated GC cells proliferation and apoptosis through p-JAK and p-STAT3 proteins. Therefore, we suspected whether miR-33a-5p would also impact on the prognosis of GC patients and the sensitivity to 5-FU through the RAP2A/JAK/STAT3 pathway.

## 2. Materials and Methods

### 2.1. Clinical Samples and Cell Lines

GC tissues and adjacent normal tissues from 30 patients were acquired at the Shanxi Cancer Hospital from 2019 to 2020. All patients signed informed consent, and the correlation sample experiments were consented by the Ethics Committee of the Shanxi Cancer Hospital.

The human SGC-7901, HGC-27, BGC-823, and MGC-803 cell lines were obtained from ATCC. They were cultured in complete medium (DMEM with 10% fetal bovine serum; Hyclone). All cells need to be replaced periodically, expand culture and cell cryopreservation, and screened for mycoplasma.

### 2.2. RNA Extraction and qQT-PCR

Total RNA was extracted by TRIZOL method (Invitrogen, NY) and converted to cDNA using the TaqMan Reverse Transcription kit (Applied Biosystems, CA) in a thermal cycler at 16°C for 25~30 minutes, 41°C for 25~30 minutes, and 85°C for 10 minutes. qRT-PCR was using the SYBR Green PCR Mix (Life Technologies, CA) at 95°C for 13 minutes, 40 cycles at 95°C for 17 seconds, and 60°C for 2 minutes. These reference genes (GAPDH) were used as control expression. The experimental data were calculated using 2^−*ΔΔ*Ct^ method. Sequence of primers: miR-33a-5p Forward: 5′-GATCCTCAGTGCATTGTAGTTGC-3′, Reverse: 5′-CTCTGTCTCTCGTCTTGTTGGTAT-3′. U6 Forward: 5′-CTCGCTTCGGCAGCACA-3′, Reverse: 5′-AACGCTTCACGAATTTGCGT-3′. RAP2A Forward: 5′-ACAATGGTGGACGAACTCTTT-3′, Reverse: 5′-CAGAACAGCATGGGTCATCTT-3′. GAPDH Forward: 5′-GGCTGTTGTCATACTTCTCATGG-3′; Reverse: 5′-AGGAAAAGCATCACCCGGAG-3′.

### 2.3. Transfection

miR-33a-5p were synthesized and transfected to GC cell lines to analyze biological function. QRT-PCR was used to determine whether the transfection efficiency was successful. Cells were divided into three group based on their specific treatment: NC group (negative control), miR-33a-5p mimics, and miR-33a-5p inhibitor. Lipofectamine 3000 (Invitrogen, USA) was used for cell transfection by the manufacturer's instructions.

### 2.4. Cell Proliferation

The cell activity was detected using the cell counting kit-8 (CCK-8) assay (Dojindo, Japan) as follows. The cells in different groups after transfection were inoculated into a 24-well plate. At 0, 1, 2, 3, and 4 days after transfection, an equal amount of GC cells were cultured into a 96-well plate. 10 *μ*L CCK-8 was then added, followed by incubation with 5% CO_2_ at 37°C for 1.5~2 h. The absorbance value (A value) at 450 nm was detected using the multifunctional microplate reader, and 630 nm was used as the reference wavelength. Cells were counted 3 times in each group, and the above assays were repeated 3 times.

### 2.5. Cell Clone Formation

In colony formation assay, the transfected cells were growing in a 6-well plate at 2 ~ 3 × 10^3^ cells per well for 10~12 days. Then, it was fixed with formaldehyde, washed with phosphate-buffered saline (PBS), stained with methyl violet, and counted the cell number using microscope (Olympus, Japan). The colony − inhibition rate = [(1 − number of experimental group)/control group] × 100%; and colony − forming rate = 1 − colony − inhibition rate.

### 2.6. Cell Invasion Assay

In the invasion assay, 2 ~ 3 × 10^5^ MGC-803 or HGC-27 cells were cultured onto the transwell chambers (Millipore, Switzerland) in serum-free medium. The Matrigel (Sigma-Aldrich, USA) was coated in the upper chamber, and the 20% FBS was added to the lower chamber. After 24 h, the cells on the surface of the chamber were removed with cotton wool. Invasive cells of the lower surface were stained with Giemsa stain (Sigma, USA) and counted with microscope (Olympus, Japan).

### 2.7. Western Blot Assay

In western blot assay, the extraction protein was separated by 10% SDS-PAGE and transferred onto PVDF membranes (Millipore, USA). Membranes were sealed with 5% nonfat dried milk and incubated with anti-RAP2A antibody (Millipore) at 1: 1500 dilution; anti-pJAK and anti-JAK antibody (Cell Signaling) at 1 : 700 dilution; anti-pSTAT3 and anti-STAT3 antibody (Cell Signaling) at 1 : 600 dilution; and anti-GAPDH antibody (Cell Signaling) at 1 : 45,000 dilution. The membranes were washed with TBST and incubated at 37°C for 2 h with second antibody (Cell Signaling) at 1 : 20,000.

### 2.8. Luciferase Reporter Assays

In luciferase reporter assays, the transfection cells were washed with PBS three times. 100 *μ*L passive lysis buffer (PLB) was added to each cell hole and collected the cell lysate. Then, add 100 *μ*L LAR II working fluid, mix it quickly, and read the first value. Add 100 *μ*L Stop & Glo® Reagent and put it into the luminescent detector to read the second value. Finally, the relative fluorescence intensity was calculated.

### 2.9. Statistical Analysis

Statistical analysis was performed with a Student's *F*-test or *t*-test. *p* < 0.05 (two-sided) was supposed significant. Data were analyzed by Prism7.02 software (La Jolla, CA, USA).

## 3. Results

### 3.1. miR-33a-5p Expression Reduced in GC Samples and Cells

**Figure 1:**
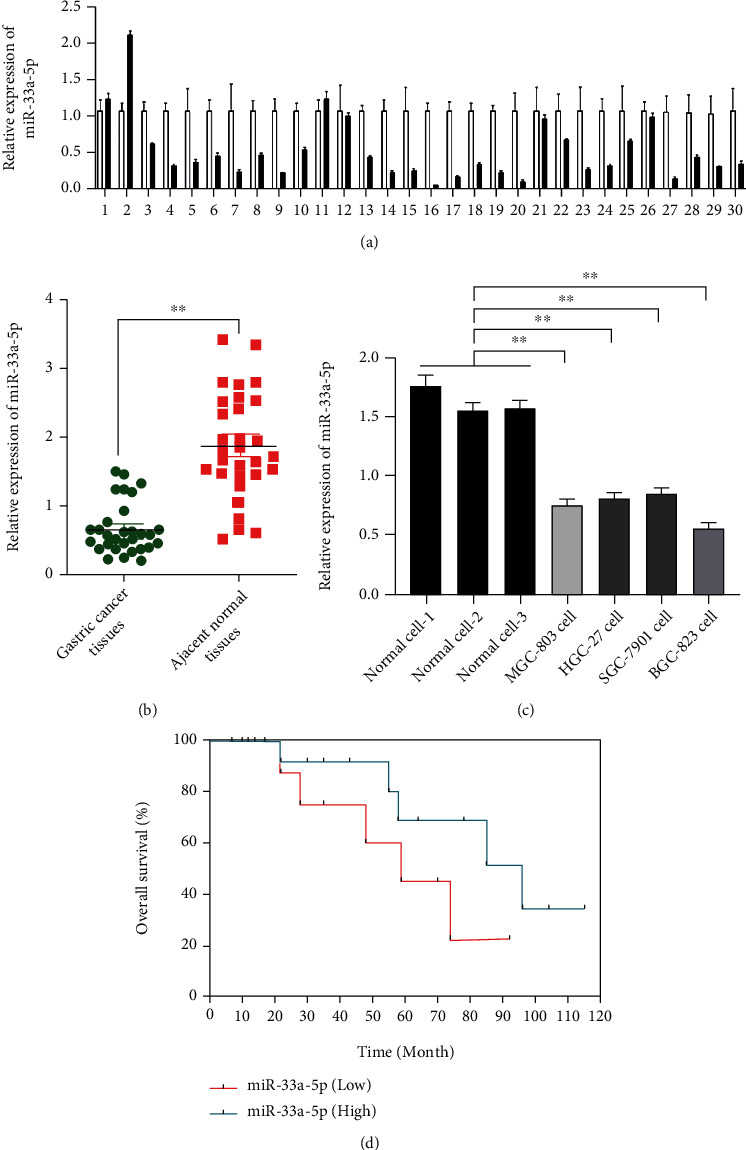
Decreased expression of miR-33a-5p in GC Samples. (a) Relative expression of miR-33a-5p in GC tissues and adjacent normal tissues pairs. (b) Mean relative expression level of miR-33a-5p in GC tissues with respect to adjacent normal tissues. (c) Endogenous relative expression level of miR-33a-5p in GC cell lines (MGC-803, BGC-823, HGC-27, and SGC-7901) and normal cells. (d) Survival analysis for miR-33a-5p in a clinical data set. miRNA levels were normalized, mean + /–SEM is shown for (a)–(c). ^∗^*p* < 0.05, ^∗∗^*p* < 0.01, *t*-test.

QRT-PCR results indicated that the expression of miR-33a-5p was lower significantly in GC tissues (Figure 1(a)). Evaluation of miR-33a-5p expression in all adjacent normal tissues showed 2.6-fold descent in GC tissues (*p* < 0.01, Figure 1(b)). The same results were got in the in vitro determination; the miR-33a-5p level was declined in GC cells (especially in BGC-823 and MGC-803cells) comparing with in normal cells (Figure 1(c)). Then, we analyzed a clinical data set with overall survival in GC tissues and showed that reduced miR-33a-5p is correlated with poor survival (Figure 1(d)).

### 3.2. MiR-33a-5p Acts as Tumor Suppressor in GC

**Figure 2:**
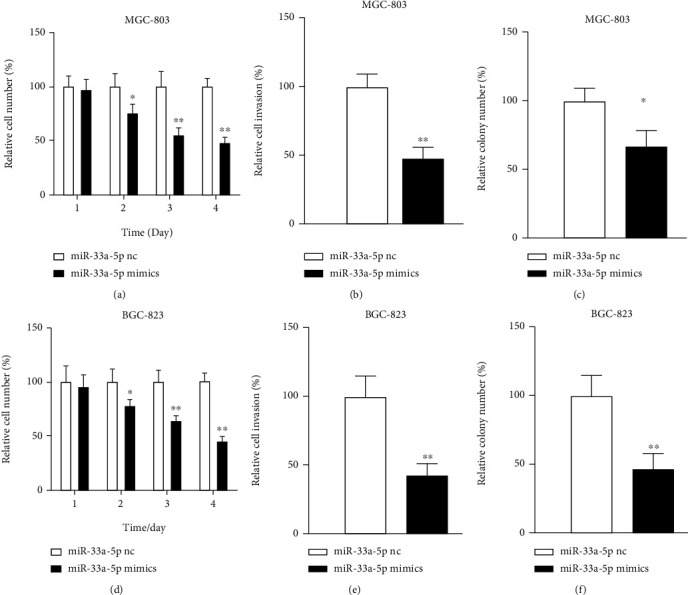
Functional impact of miR-33a-5p mimics. (a) Proliferation, (b) invasion, and (c) anchorage independent growth of MGC-803 cells transfected with miR-33a-5p mimic. (d) Proliferation, (e) invasion, and (f) anchorage independent growth of BGC-823 cells transfected with miR-33a-5p mimic. Mean + /–SEM is shown. ^∗^*p* < 0.05, ^∗∗^*p* < 0.01; *t*-test.

**Figure 3:**
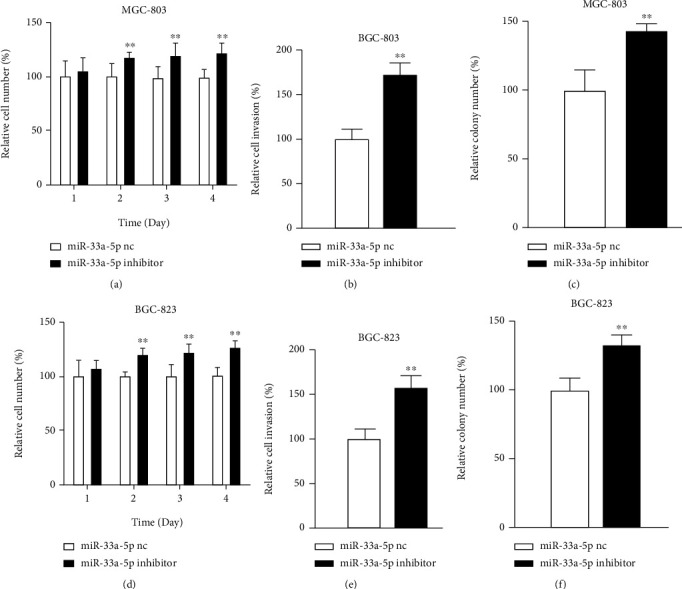
Functional impact of miR-33a-5p inhibition. (a) Proliferation, (b) invasion, and (c) anchorage independent growth of MGC-803 cells transfected with miR-33a-5p inhibitor. (d) Proliferation, (e) invasion, and (f) anchorage independent growth of BGC-823 cells transfected with miR-33a-5p inhibitor. Mean + /–SEM is shown. ^∗^*p* < 0.05, ^∗∗^*p* < 0.01; *t*-test.

To analyze what impact miR-33a-5p plays in GC cells, we transfected MGC-803 and BGC-823 cells with miR-33a-5p nc or miR-33a-5p mimics/inhibitor and evaluated the alterations in cell functions. In cell assays, miR-33a-5p mimics transfected in MGC-803 cells proliferation descended by up to 51%, invasion by 52%, and clone formation by up to 33% (Figures 2(a)–2(c)). The result was the same in BGC-823 cells, and miR-33a-5p overexpression decreased the proliferation by 55%, invasion by 58%, and clone formation by 53% (Figures 2(d)–2(f)). Then, we further analyzed the effectiveness of miR-33a-5p inhibitor transfection in cells. MiR-33a-5p inhibitor heightened proliferation by 22%, invasion by 72%, and clone formation by 43% (Figures 3(a)–3(c)). In BGC-823 cells, miR-33a-5p inhibitor ascended the proliferation by 27%, invasion by 58%, and clone formation by 33% (Figures 3(d)–3(f)). Taken together, these experiments reveal that miR-33a-5p has tumor suppression functions in GC cells.

### 3.3. Identification of miR-33a-5p Targets in GC

**Figure 4:**
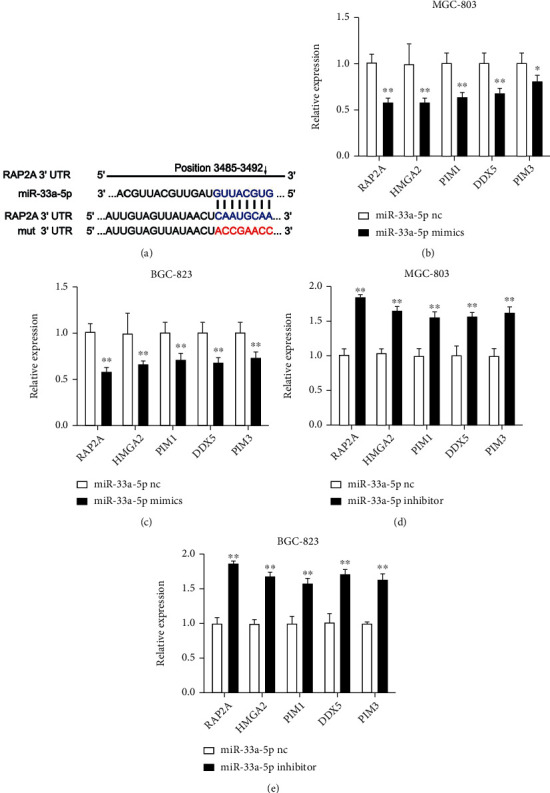
MiR-33a-5p target genes. (a) The target-map representation of relationship between in miR-33a-5p and RAP2A. (b) QRT-PCR validation of selected potential miR-33a-5p target genes in miR-33a-5p mimics transfected (b) MGC-803 and (c) BGC-823 cells and in miR-33a-5p inhibitor transfected (d) MGC-803 and (e) BGC-823 cells compared to the control cells. mRNA levels were normalized to GAPDH. Mean + /–SEM is shown for (b)–(e). ^∗^*p* < 0.05, ^∗∗^*p* < 0.01; *t*-test.

To explore potential miR-33a-5p targets in GC cells, we chose the website (https://www.targetscan.org/vert_80/) to identify possible binding sites (Figure 4(a)). The analysis revealed a conserved sequence between positions 3485-3492 of RAP2A 3′UTR as a potential target for miR-33a-5p. We prioritized these potential target genes and screened 5 candidate genes of miR-33a-5p. Then, we selected those genes to further validation: RAP2A, HMGA2, PIM1, DDX5, and PIM3. In BGC-823 and MGC-803 cells, the increased expression of miR-33a-5p reduced these genes significantly, especially RAP2A (Figures 4(b) and 4(c)). However, miR-33a-5p downregulation confirmed RAP2A, HMGA2, PIM1, DDX5, and PIM3 as potential targets in BGC-823 and MGC-803 cells (Figures 4(d) and 4(e)). According to the results of qRT-PCR, RAP2A was selected for further analysis, because it declined significantly after changes in the miR-33a-5p expression.

### 3.4. MiR-33a-5p Downregulates RAP2A through Targeting RAP2A

**Figure 5:**
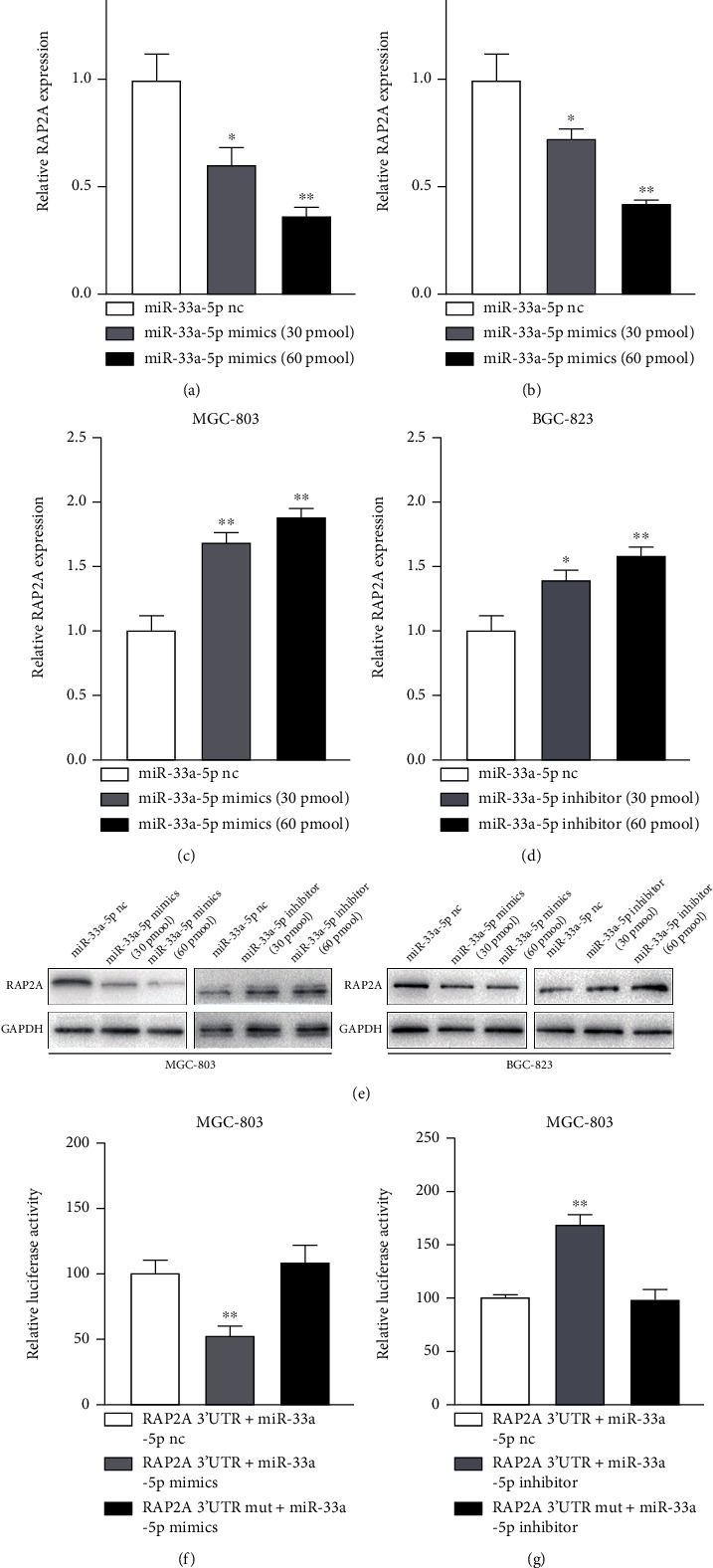
RAP2A expression is repressed by 3′UTR binding of miR-33a-5p. Relative mRNA level of RAP2A in MGC-803 cells transfected with either 30 pmol or 60 pmol (a) mimic miR-33a-5p or (c) miR-33a-5p inhibitor. Relative mRNA level of RAP2A in BGC-823 cells transfected with either 30 pmol or 60 pmol (b) miR-33a-5p mimic or (d) miR-33a-5p inhibitor. (e) Relative protein level of RAP2A in MGC-803 cells and BGC-823 transfected with either 30 pmol or 60 pmol miR-33a-5p mimics or miR-33a-5p inhibitor. Relative luciferase activity of wild-type and mutated RAP2A 3′UTR in MGC-803 cells transfected with (f) miR-33a-5p mimic or (g) miR-33a-5p inhibitor. mRNA levels were normalized to GAPDH and protein levels were normalized to GAPDH, Mean + /–SEM is shown. ^∗^*p* < 0.05, ^∗∗^*p* < 0.01.

To validate the targets RAP2A, we initially selected 2 different concentrations of mature miR-33a-5p inhibitor or mimics to analyze RAP2A mRNA and protein levels. QRT-PCR and western blot results showed that the RAP2A expression decreased significantly with the higher concentrations of miR-33a-5p mimics (Figures 5(a), 5(b), and 5(e)). On the contrary, the higher miR-33a-5p inhibitor resulted in RAP2A expression increased obviously (Figures 5(c)–5(e)).

Cotransfecting with miR-33a-5p mimics/inhibitor and either the wild-type RAP2A 3′UTR or the mutation RAP2A 3′UTR (same region in the predicted seed sequence) containing luciferase reporter in MGC-803 cells revealed that miR-33a-5p blocked the luciferase activity of the wild-type RAP2A 3′UTR, however, not that of the RAP2A 3′UTR mut compared to miR-33a-5p nc control (Figure 5(f)). Furthermore, cotransfection with miR-33a-5p inhibitor led to increase in the luciferase activity of the wild-type RAP2A 3′UTR obviously, but no alter was founded in the mutant reporter construct activity (Figure 5(g)). According to the results, RAP2A targets miR-33a-5p directly in MGC-803 cell lines.

### 3.5. Aberrant Expression of RAP2A in GC Tissues and Cell Lines

**Figure 6:**
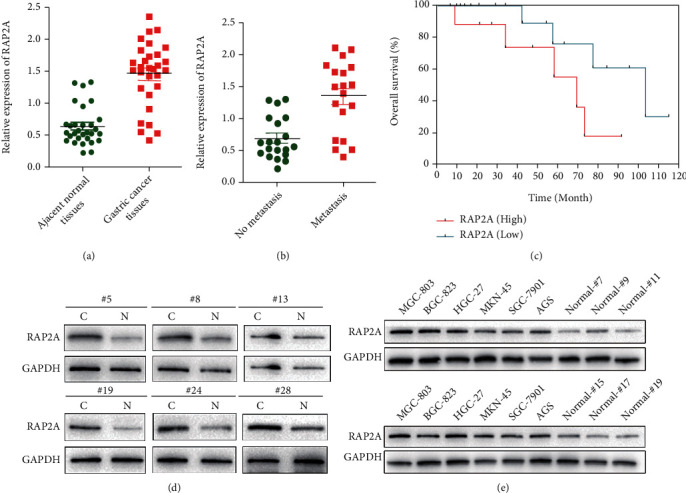
Decreased expression of RAP2A in GC Samples. (a) Relative expression of RAP2A in GC tissues and adjacent normal tissues pairs. (b) Relative expression of RAP2A in no metastasis and metastasis pairs. (c) Survival analysis for RAP2A in a clinical data set. Relative protein level of RAP2A in different samples (d) and cells (MGC-803, BGC-823, HGC-27, MKN-45, AGS, and SGC-7901and normal cells) (e). mRNA levels were normalized, Mean + /–SEM is shown for (a) and (b). ^∗^*p* < 0.05, ^∗∗^*p* < 0.01, *t*-test.

We performed qPCR analysis in 30 pairs of clinical GC tissues to detect RAP2A expression. The results showed that RAP2A mRNA levels were elevated in 26 cases (87%) of GC tissues (C) compared with their adjacent normal tissues (N), but only 4 tissues (13%) showed downregulated RAP2A mRNA levels (Figure 6(a)). To further explore the correlation between RAP2A and clinicopathological factors, we analyzed the relative RAP2A levels in GC tissues at different stages of progression (nonparametric test). Obviously, we got that a high RAP2A level accompanied with GC pM stage (Figure 6(b), nonmetastasis vs. metastasis, *p* < 0.001) in GC patients. In addition, GC patients with high RAP2A expression had worse disease-free survival and overall survival (Figure 6(c)).

To further confirm these results, we detected the RAP2A protein levels in 6 pairs of GC tissues (C and N) randomly. The protein level of RAP2A was significantly elevated (>2-fold) in GC tissues (Figure 6(d)). Moreover, The RAP2A protein was sharply rose in GC cell lines (MKN-45, MGC-803, HGC-27, BGC-823, AGS, and SGC-7901) in contrast with normal cells (Figure 6(e)). The results showed that the upregulation of RAP2A may play a potential driving role in GC tumorigenesis.

### 3.6. MiR-33a-5p Reverses the Effects of RAP2A Associated with GC Progression

**Figure 7:**
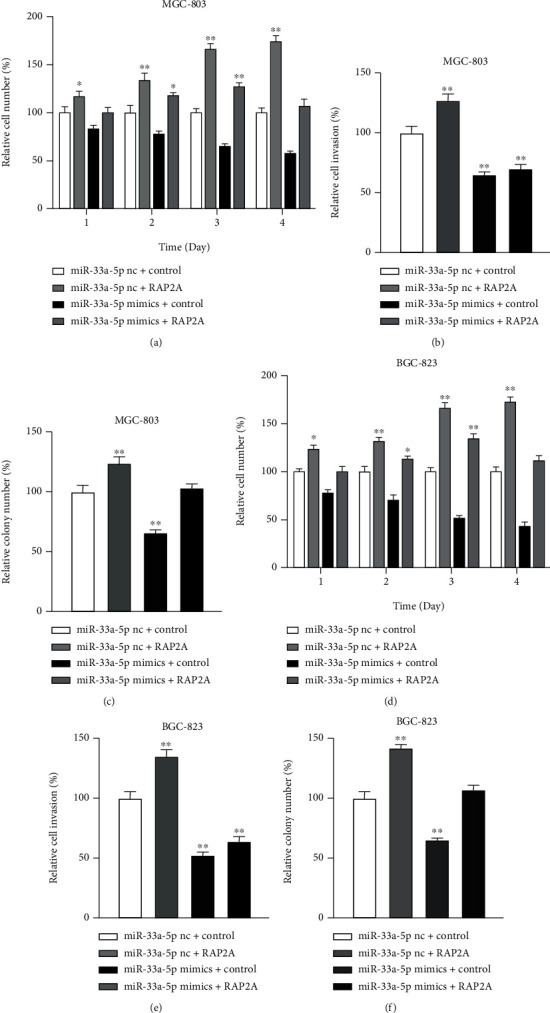
RAP2A expression increases proliferation and anchorage independent growth. (a) Proliferation, (b) invasion, and (c) colony number and RAP2A overexpressing MGC-803 cells transfected with miR-33a-5p mimic. (d) Proliferation, (e) invasion, and (f) colony number and RAP2A overexpressing BGC-823 cells transfected with miR-33a-5p inhibitor. Mean + /–SEM is shown. ^∗^*p* < 0.05, ^∗∗^*p* < 0.01; *t*-test.

RAP2A is a member of the small GTPase protein superfamily, which is involved in many cellular functions including cell adhesion and proliferation. In our studies, miR-33a-5p mimics led to a decline in RAP2A mRNA and protein levels (Figures 5(a) and 5(e)). In MGC-803 cells, RAP2A overexpression promoted proliferation by 74% and cell invasion by 25% (Figures 7(a) and 7(b)). However, the proliferation induction rate of RAP2A was reduced to 6%, and the clone formation rate was reduced to 4% after the introduction of miR-33a-5p into MGC-803 cells (Figures 7(a) and 7(c)). In BGC-823 cells, the cell proliferation decreased from 72% to 8%, and the clone formation rate decreased from 34% to 7% after the introduction of miR-33a-5p (Figures 7(d)–7(f)).

### 3.7. RAP2A Reduces the Sensitivity of GC Cells to 5-FU through the JAK/STAT3 Signaling Axis

**Figure 8:**
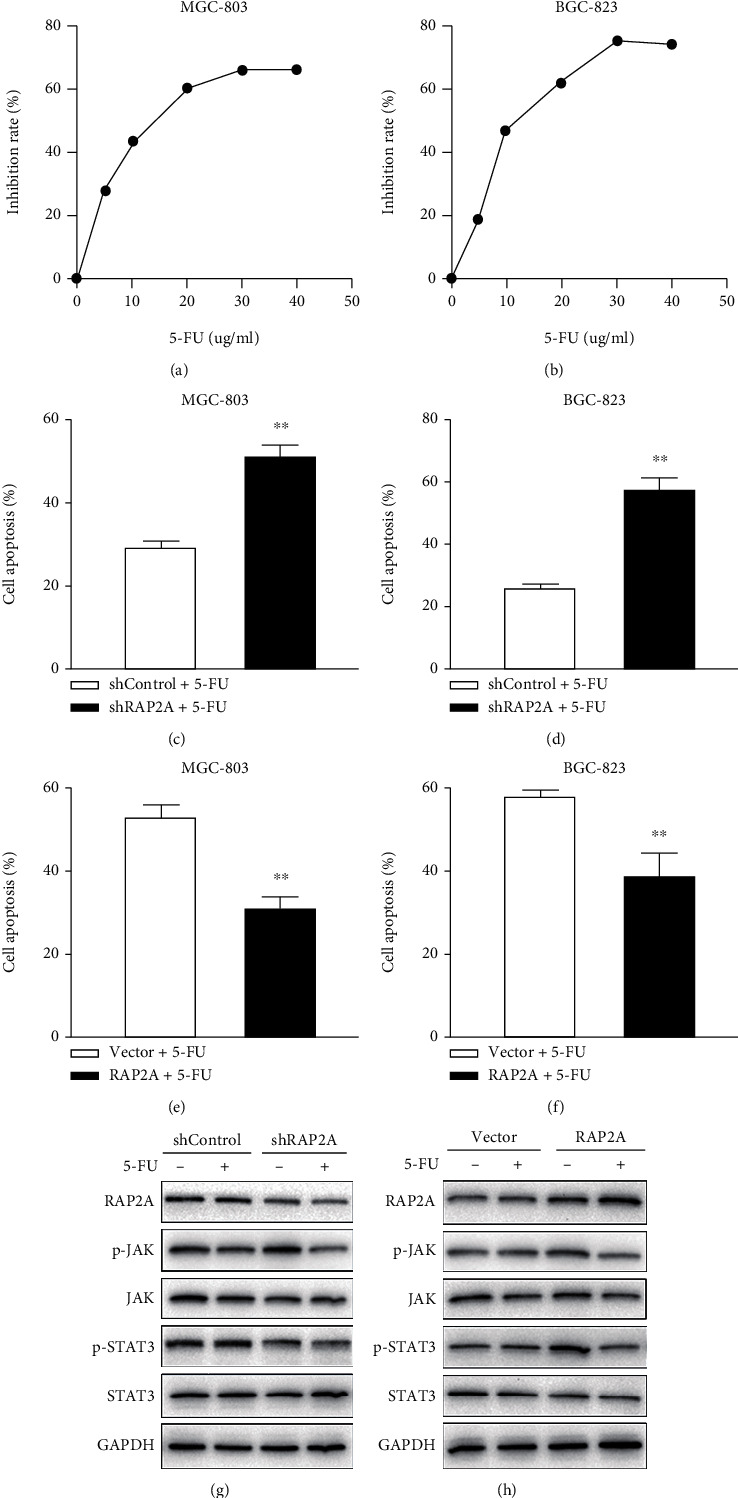
Knockdown or overexpression of RAP2A affects the sensitivity of GC cancer cells to 5-FU. 24 h after drug administration, CCK-8 was added, the IC50 of MGC-803 cells was 11.134 *μ*g/mL (a), and that of BGC-823 cells was 10.634 *μ*g/mL (b). Cell viability was detected by CCK-8. (c) MGC-803 shControl and shRAP2A were treated with 11 *μ*g/mL 5-FU, and (d) BGC-823 shControl and shRAP2A were treated with 10 *μ*g/mL 5-FU. (e) MGC-803 vector and RAP2A were treated with 11 *μ*g/mL 5-FU, and (f) BGC-823 vector and RAP2A were treated with 10 *μ*g/mL 5-FU. (g) Representative western blot image shows the expression of RAP2P, pJAK, JAK, pSTAT3, and STAT3 in MGC-803 shControl and MGC-803 shRAP2P cells treated with 11 *μ*g/mL 5-FU for 24 h. (h) Representative western blot image shows the expression of RAP2P, pJAK, JAK, pSTAT3, and STAT3 in BGC-823 vector and BGC-823 RAP2P cells treated with 10 *μ*g/mL 5-FU for 24 h.

MGC-803 cells and BGC-823 cells of wild-type cells were treated with different concentrations of 5-FU. 24 h after drug administration, CCK-8 was added, and OD value was detected by a microplate reader. The IC50 of MGC-803 cells was 11.134 *μ*g/mL (Figure 8(a)) and that of BGC-823 cells was 10.634 *μ*g/mL (Figure 8(b)). To explore whether RAP2A affects on 5-FU-induced apoptosis of GC cells, cells were collected after 5-FU treatment for 24 h, and cell apoptosis ratio was detected by flow cytometry. The results showed that the apoptosis rate of MGC-803 shControl was 29.06% and that of MGC-803 shRAP2A was 51.13% (Figure 8(c)). Low RAP2A expression significantly increased the 5-FU-induced apoptosis rate (*p* < 0.01). The apoptosis rate of MGC-803 no-load cells was 53.42% and that of MGC-803 RAP2A was 31.45% (Figure 8(e)). The high expression of RAP2A significantly reduced the 5-FU-induced apoptosis rate (*p* < 0.01). The results showed that the apoptosis rate of BGC-823 shControl was 25.13% and that of BGC-823 shRAP2A was 57.04% (Figure 8(d)). Low RAP2A expression significantly increased the 5-FU-induced apoptosis rate (*p* < 0.01). The apoptosis rate of BGC-823 no-load cells was 57.12% and that of BGC-823 RAP2A was 38.24% (Figure 8(f)). The high expression of RAP2A significantly reduced the 5-FU-induced apoptosis rate (*p* < 0.01).

Studies have shown that RAP2A promotes the proliferation of GC cells by activating the JAK/STAT3 signaling pathway. To investigate the mechanism of RAP2A affecting the sensitivity of GC cells to 5-FU, we detected the phosphorylation level of JAK and the STAT3. The results indicated that the expression levels of p-JAK and p-STAT3 in MGC-803 shRAP2A cells were significantly decreased 24 h after 5-FU treatment compared with MGC-803 shControl cells (*p* = 0.023; *p* = 0.005) (Figure 8(g)). Compared with BGC-823 no-load cells, the levels of p-JAK and p-STAT3 in BGC-823 RAP2A cells were significantly increased (*p* = 0.004; *p* = 0.006) (Figure 8(h)). These results suggest that RAP2A plays a role in reducing the sensitivity of GC cells to 5-FU by upregulating the JAK/STAT3 pathway.

## 4. Discussion

In our conclusion, we determined that the expression of miR-33a-5p was decreased in GC tissues compared with adjacent normal tissues. In addition, miR-33a-5p overexpression increased the proliferation, invasion, and clone growth of GC cells in vitro, while reduction of miR-33a-5p had the inverse effect. Therefore, our results suggest that miR-33a-5p plays a tumor suppressor role in GC. Moreover, we induced significant changes in miR-33a-5p levels through enhancing or silencing in cell experiments. However, as the dose response of miR-33a-5p deletion in GC is not clear, it is difficult to determine its corresponding biological response in GC.

Many studies have found that miR-33a-5p plays a regulatory role in variety of tumors [12, 16]. To explore the miR-33a-5p effect in GC cells, we confirmed the expression, potential targets by QRT-PCR, western blot, and luciferase assay. Analysis indicated that HMGA2, PIM1, DDX5, PIM3, and RAP2A were potential targets of miR-33a-5p. Since RAP2A is an obvious oncogene in GC cells, we selected it for further experimental [4].

As we mentioned above, miR-33a-5p could regulate multiple genes in GC cell lines; in this study, upregulation of RAP2A almost promotes cell proliferation and invasion in GCs, suggesting that RAP2A is the dominant target of miR-33a-5p effect in the GC cells. The relationship between RAP2A and prognosis and progression of GC was further investigated. To further explore the correlation between RAP2A and clinicopathological factors, we analyzed the relative RAP2A levels in GC tissues at different stages of progression (nonparametric test). Obviously, we got that a high RAP2A level accompanied with GC pM stage (Figure 6(b), nonmetastasis vs. metastasis, *p* < 0.001) in GC patients. In addition, GC patients with high RAP2A expression had worse disease-free survival and overall survival.

Drug resistance based on 5-FU is a major problem in the clinical treatment of advanced GC. It has not been reported whether the high expression of RAP2A in GC will affect the sensitivity of GC cells to 5-FU. Studies have shown that RAP2A promotes the proliferation of GC cells by activating the JAK/STAT3 signaling pathway. To investigate the mechanism of RAP2A affecting the sensitivity of GC cells to 5-FU, we detected the phosphorylation level of JAK and STAT3. The results indicated that the expression levels of p-JAK and p-STAT3 in MGC-803 shRAP2A cells were significantly decreased 24 h after 5-FU treatment compared with MGC-803 shControl cells (*p* = 0.023; *p* = 0.005). The results of this study showed that RAP2A negatively regulated the sensitivity of GC cells to 5-FU. This suggests that RAP2A may be a biomarker for predicting the effectiveness of 5-FU-based treatment decisions.

In summary, our results indicate that miR-33a-5p is underexpressed in GC cells, and miR-33a-5p negatively regulates RAP2A. However, the high expression of RAP2A was negatively correlated with the survival of GC patients, and the high expression of RAP2A could reduce the sensitivity of GC cells to 5-FU. RAP2A is expected to be a marker of prognosis and chemotherapy resistance in patients with GC.

## Data Availability

The underlying data supporting the results of your study can be found by lifenglover@sina.com.

## Abbreviations

GC: Gastric cancer
RAP2A: Ras-related protein rap-2a
HMGA2: High mobility group AT-hook 2
PIM1: Pim-1 proto-oncogene
DDX5: DEAD-box helicase 5
PIM3: Pim-3 proto-oncogene
miRNA: microRNA.
